# Freedom Restrictive Coercive Measures in Forensic Psychiatry

**DOI:** 10.3389/fpsyt.2020.00146

**Published:** 2020-03-05

**Authors:** Erich Flammer, Udo Frank, Tilman Steinert

**Affiliations:** ^1^Center for Psychiatry Suedwuerttemberg, Clinic for Psychiatry and Psychotherapy I, Ulm University, Ravensburg, Germany; ^2^Center for Psychiatry Suedwuerttemberg, Ravensburg, Germany

**Keywords:** coercion, forensic psychiatry, seclusion, restraint, involuntary medication, register data

## Abstract

**Background:** In Germany, people suffering from severe mental illness who have committed serious offenses and have considerably reduced or suspended criminal responsibility can be detained and treated in forensic psychiatric hospitals. In the German federal state of Baden-Wuerttemberg, all psychiatric hospitals including forensic psychiatric hospitals are obliged to record data on every coercive intervention and to submit them to a central registry. The objective of this study was to determine key measures for the use of seclusion and restraint and to compare them with data from the same registry on the use of coercion in general inpatient mental health care.

**Methods:** Data on the main psychiatric diagnosis according to ICD-10, type and duration of each coercive measure and number of treated cases according to diagnoses, and cumulated number of days of treatment from all 8 forensic facilities in the state of Baden-Wuerttemberg covering a catchment area with about 11 million inhabitants were collected at the treated-case-level for 3 years.

**Results:** 22.6% of the cases treated in 2017 in forensic psychiatric hospitals were subjected to seclusion, and 3.8% were subjected to mechanical restraint. The mean cumulated duration of seclusion episodes per affected case was 343.9 h and the mean cumulated duration of restraint episodes was 261.7 h. 13.2% of the treated cases were subjected to room confinement with a mean cumulated duration of 539.1 h per affected case. Involuntary medication was applied in 1.9% of the cases. In general psychiatry, 2.9% of the treated cases were subjected to seclusion, and 4.7% were subjected to mechanical restraint. The mean cumulated duration per affected case amounted to 32.2 h for seclusion episodes and to 37.6 h for restraint episodes. Involuntary medication was applied in 0.6% of cases.

**Conclusion:** Compared to general psychiatry, mechanical restraint is used in forensic psychiatry substantially less frequently and seclusion substantially more frequently. Room confinement is used only in forensic psychiatric hospitals. Use of involuntary medication is rare. On the one hand, recorded involuntary medication comprises only clear actions against the patient's expressed will as defined by law. Psychological pressure to take medication to avoid other forms of coercion and to achieve higher levels of freedom within the facility is not recorded. On the other hand, the low numbers of clear involuntary medication probably reflect the high legal threshold for such interventions, and, consequently, efforts by staff to motivate voluntary acceptance. The long duration of freedom-restricting coercive measures in forensic psychiatry probably reflects the selection of patients at high risk of violence.

## Introduction

In Germany, people suffering from severe mental illness who have committed offenses and are deemed to have considerably reduced or suspended criminal responsibility at the time of the offense can be detained in forensic psychiatric hospitals according to penal law (§63 German penal law). A criminal court can order the detention of an individual in a forensic psychiatric hospital, if due to his or her condition, the individual presents a significant risk of committing harmful or dangerous acts, and the detention will be suspended only when the court is of the opinion that the individual presents no future danger to the public or that ongoing detention is no longer proportionate. A similar rule applies to offenders with addictive disorders who can be detained in specialized forensic units (§64 German Criminal Law); however, in these cases, the maximum duration is limited to 2 years plus two-thirds of a parallel prison sentence.

The conditions of forensic psychiatric treatment in the respective facilities are regulated by the federal law of the 16 federal states, with certain differences between them. This applies also to the use of freedom-restrictive coercive interventions. The law explicitly mentions seclusion and mechanical restraint, as well as being confined in one's own room (room confinement). With regard to involuntary medication, the threshold is high since a seminal decision by the Federal Constitutional Court (Bundesverfassungsgericht) in 2011 (1). Except for acute emergencies, administering medication against a patient's will is only possible after an expert review and an additional judge's decision for a limited period of time. Involuntary treatment can only be allowed in cases of impaired decision-making capacity, to prevent serious harm of the patient or other persons, to re-establish free decision-making capacity and inclusion into a community, after intensive attempts to persuade the patient, and if the expected advantages of treatment are expected to outweigh possible negative effects. Serious concern has been expressed that these high requirements sometimes lead to a very long duration of legal procedures and involuntary treatment is frequently refused or only allowed for a very limited period.

As a consequence, experts claim that since the legal adoption of the Constitutional Court's decisions, seclusion is used for an overly long duration and often due to a lack of appropriate treatment for psychotic disorders (2). However, in practice there seems to be some variation among the responsible courts according to anecdotal evidence.

In the German federal state of Baden-Wuerttemberg, the Mental Health Law was adopted following the decisions of the Federal Constitutional Court in 2015. A unique feature of this law is a ruling requiring the collection of data on seclusion, restraint, room confinement (which happens only in forensic psychiatry), emergency medication, and involuntary medication following a judge's decision in all psychiatric hospitals, including forensic psychiatry. It is mandatory for all psychiatric hospitals to supply this data to acentral register.

The central recording of coercive measures, on the one hand, makes special demands on data protection and data security in view of the highly sensitive personal data. On the other hand, the simplest possible transmission of the data to be delivered is desirable. Therefore, an online platform was set up after detailed consultation with and approval of the State Data Privacy and Data Security Officer. The platform serves both for uploading data by the institutions and for downloading data by the evaluation office. Thus, the register offers the unique possibility to analyze the use of coercive interventions in all eight forensic psychiatric hospitals in a Federal State with 11 million inhabitants. The patient population of these hospitals is very well-characterized by other data available from the Forensic Base Documentation (FoDoBa) (3) which has been in use in Baden-Wuerttemberg since 2009.

The objective of this study was to determine key measures for the use of seclusion, restraint, and involuntary medication as defined earlier in non-forensic patients (4) and to compare them with the practice in forensic patients.

## Materials and Methods

### Data Sources

We obtained data on patients and on coercive measures from two sources: the central register (CR) (5) and the Forensic Base Documentation (FoDoBa) (3) which is run separately from the central register by the forensic facilities. Due to data privacy, the CR and the FoDoBa cannot be merged. From the FoDoBa only aggregated data was available. Data from the CR was available for the years 2015 to 2017. Data from the FoDoBa was available for the years 2009 to 2017.

### Data Recording and Data Structure in the FoDoBa

The FoDoBa was introduced by all forensic hospitals in Baden-Wuerttemberg in 2009 and contains extensive data on age, gender, socioeconomic status, number and kind of offenses, personal history, living conditions, family situation, and medical and psychiatric history of all detained patients. In 2009 and 2010, data had been provided twice a year, since 2011 data is provided on a yearly basis.

### Data Recording and Data Structure in the Central Register

The CR contains data on coercive interventions from both forensic psychiatry and general psychiatry. Each facility with an obligation to report data has its own protected upload area on the online platform of the CR. When uploading data, a comparison between the user name and mandatory fields for hospital identification is made. In the case of contradictions, the data upload is blocked with a simultaneous warning to the user. There are three datasets to be uploaded. Dataset 1 contains all the coercive measures to be reported, together with the hospital identification code, pseudonymized case number, postal code of residence, gender, main diagnosis, legal basis for hospital stay at the beginning of the coercive measure, type, and duration of coercive measure. The other two datasets contain aggregated data on the number of treated cases and days of treatment. According to the requirements set by the State Data Privacy and Data Security Officer, the data of the CR is structured in such a way that the identification of specific persons is not possible, i.e., the data is anonymized. This especially applies to Dataset 1. Evaluations based on the central register are provided regularly to the psychiatric and forensic hospitals and to the parliament of Baden-Wurttemberg.

### Definitions of Seclusion Episodes, Restraint Episodes, and Room Confinement

Seclusion is defined as separation into a specially secured room which can be locked from the outside. The affected patient is brought into a separate room and locked up there or prevented from leaving the room.

Restraint is defined as physical restriction of movement by belts (4).

Room confinement means securing a patient in his or her own room. In contrast to seclusion, it is not a special, safe (and mostly empty) room and in contrast to time-out, the room is locked from the outside.

### Case Definition in the CR

In the CR for non-forensic patients in general psychiatric hospitals, each complete patient treatment episode within a given reporting year is defined as a treatment case. If, for example, a patient had been admitted on December 15, 2016 and was discharged on January 10, 2017, he or she is counted in the reporting year of 2017 with all 26 days of treatment in 2016 and 2017. If a patient had been admitted on December 20, 2017 and was discharged on January 5, 2018, she is not counted in 2017.

For forensic patients each newly started, ongoing, or completed patient's treatment episode within a given reporting year is defined as a treatment case and is tallied in this reporting year. Different to non-forensic patients, in forensic patients only those days of treatment which accrued within a given reporting year are considered. This difference arises because the majority of forensic patients are not discharged within a given year and their total length of stay is still unknown.

As no patient identification is given and case identification numbers are pseudonymized, it is not possible to check whether two or more cases relate to the same patient. This applies to both non-forensic patients and forensic patients.

### Definition of Treated Cases in the FoDoBa

The FoDoBa defines treated cases as the number of patients being treated as inpatients on a given day (i.e., December 31).

### Data Analysis

The data analysis was carried out at case level. Therefore, a treatment case may have several different coercive measures and may be tallied in all categories, a case with seclusion episode, a case with restraint episode, and a case with room confinement. Due to the structure of the data, the identification of specific persons was not possible.

### Ethical Considerations

The Ethics Committee of Ulm University waived the requirement for ethics approval as approval is not required for studies analyzing anonymized data, in accordance with national legislation and institutional requirements.

## Results

In 2017, eight forensic hospitals and 32 non-forensic hospitals reported data to the central register. In the eight forensic hospitals, on December 31, 2017, the number of treated patients amounted to 1,049. Of these, 131 patients were preliminarily committed following a crime and awaiting trial, and 918 had been given a hospital order by court decision. Most of the patients suffered from psychotic disorders or severe personality disorders (Table 1), and 362 had an addictive disorder. German law allows detention for people with addictive disorders who have committed a crime of up to 2 years in a specialized forensic psychiatric unit. 42.0% of all patients had a migration background. 18.3% had not completed high school education, 4.7% had attended schools for children with learning disabilities, 64.2% had 9 or 10 years of education. Of the 918 convicted patients, 64 (7.0%) had been convicted for murder or manslaughter, 115 (12.5%) for attempted murder, 252 (27.5%) for assault and battery, and 47 (5.1%) for a sexual offense.

**Table 1:** Treated cases in the forensic and the non-forensic hospitals.

	**Number of treated cases (%)**			
	**Forensic hospitals**			**Non-forensic hospitals**
**Main diagnosis**	**2015**	**2016**	**2017**	**2017**
F0/G30	43 (3.2%)	39 (2.7 %)	35 (2.4%)	9,387 (8.2%)
F1	514 (38.0%)	596 (40.9%)	596 (41.6%)	31,549 (27.4%)
F2	560 (41.4%)	578 (39.7%)	568 (39.7%)	19,159 (16.7%)
F3	28 (2.1%)	30 (2.1%)	28 (2.0%)	30,385 (26.4%)
F4	*	*	*	10,262 (8.9%)
F5	*	*	*	694 (0.6%)
F6	105 (7.8%)	113 (7.8%)	116 (8.1%)	4,574 (4.0%)
F7	63 (4.7%)	70 (4.8%)	63 (4.4%)	806 (0.7%)
F8	10 (0.7%)	8 (0.5%)	9 (0.6%)	276 (0.2%)
F9	11 (0.8%)	11 (0.8%)	*	1,932 (1.7%)
Other	15 (1.1%)	6 (0.4%)	7 (0.5%)	5,987 (5.2%)
Total	1,352	1,456	1,431	115,011

*Omitted due to small numbers in order to ensure data privacy.

In the eight forensic hospitals 1,431 patients were treated in 2017 (Table 1) with a total of 365,341 days of treatment. Three hundred and twenty four cases (22.6%, Table 2) were subjected to 9,358 seclusion episodes and 54 cases (3.8%) were subjected to 703 restraint episodes (Table 3). The mean cumulated duration of seclusion episodes per affected case was 343.9 h (median = 90.8, Table 4), and the mean cumulated duration of restraint episodes was 261.7 h (median = 26.7, Table 5). If cases with a cumulated duration of both seclusion and restraint episodes of more than 3,000 h are excluded, the mean cumulated duration is 204.3 h for seclusion episodes and 201.8 for restraint episodes. One hundred and eighty nine cases (13.2%) were subjected to room confinement in 2017. The mean cumulated duration per affected case was 539.1 h. The duration of the respective coercive intervention in relation to the total duration of hospital stay was 1.3% for seclusion episodes, 0.2% for restraint episodes, and 1.2% for room confinement. Involuntary medication was applied in 27 cases (1.9%, Table 6). The mean number of all coercive measures per bed per year was 10.1, 9.3 for seclusion and 0.7 for restraint. The mean number of involuntary medications was 0.02 for emergency medication and 0.02 for medication according to a court order.

**Table 2:** Cases with seclusion episodes.

	**2015**		**2016**		**2017**	
**Main diagnosis**	**Forensic hospitals (%)**	**General psychiatric hospitals (%)**	**Forensic hospitals (%)**	**General psychiatric hospitals (%)**	**Forensic hospitals (%)**	**General psychiatric hospitals (%)**
F0/G30	20.9	3.6	35.9	3.5	37.1	3.7
F1	9.5	1.7	11.7	1.9	11.7	2.0
F2	26.4	7.8	28.9	7.3	29.0	7.1
F3	35.7	1.1	23.3	1.2	21.4	1.1
F4	100.0	1.1	50.0	1.5	0.0	1.8
F5	0.0	0.5	0.0	0.8	0.0	0.6
F6	21.0	3.5	28.3	4.1	28.4	4.4
F7	28.6	6.7	38.6	14.4	44.4	17.9
F8	70.0	7.1	62.5	5.3	44.4	6.9
F9	27.3	3.7	36.4	3.3	50.0	3.3
Other	26.7	0.6	33.3	0.7	25.0	0.3
Total	20.1	2.9	22.7	3.0	22.6	2.9

**Table 3:** Cases with restraint episodes.

	**2015**		**2016**		**2017**	
**Main diagnosis**	**Forensic hospitals (%)**	**General psychiatric hospitals (%)**	**Forensic hospitals (%)**	**General psychiatric hospitals (%)**	**Forensic hospitals (%)**	**General psychiatric hospitals (%)**
F0/G30	2.3	14.3	5.1	14.2	5.7	12.6
F1	0.6	3.4	0.5	3.4	1.2	3.9
F2	5.0	9.7	5.0	9.9	5.5	9.9
F3	10.7	1.6	0.0	1.4	3.6	1.5
F4	50.0	1.5	50.0	1.8	0.0	1.9
F5	0.0	1.6	0.0	1.5	0.0	2.0
F6	3.8	5.2	7.1	5.3	3.4	6.5
F7	12.7	9.6	7.1	10.9	9.5	11.4
F8	10.0	5.8	50.0	3.1	22.2	3.3
F9	0.0	1.7	18.2	2.1	0.0	2.0
Other	0.0	2.0	16.7	1.5	12.5	0.4
Total	3.6	4.8	3.8	4.9	3.8	4.7

**Table 4:** Cumulated duration of seclusion episodes.

	**Cases with seclusion**		**Mean (median) cumulated duration of seclusion episodes per affected case (hours)**	
	**Forensic hospitals (%)**	**General psychiatric hospitals (%)**	**Forensic hospitals**	**General psychiatric hospitals**
2015	20.1	2.9	369.1 (74.6)	34.6 (12.0)
2016	22.7	2.9	375.2 (85.0)	40.5 (11.3)
2017	22.6	2.9	343.9 (90.8)	32.2 (10.0)

**Table 5:** Cumulated duration of restraint episodes.

	**Cases with restraint**		**Mean (median) cumulated duration of restraint episodes per affected case (hours)**	
	**Forensic hospitals (%)**	**General psychiatric hospitals (%)**	**Forensic hospitals**	**General psychiatric hospitals**
2015	3.6	4.8	233.2 (44.1)	40.3 (11.8)
2016	3.8	4.9	413.6 (30.6)	39.0 (11.3)
2017	3.8	4.7	261.7 (26.7)	37.6 (10.9)

**Table 6:** Cases with involuntary medication.

	**Cases with involuntary medication**		**Cases with emergency medication**		**Cases with medication according to a court order**	
	**Forensic hospitals (%)**	**General psychiatric hospitals (%)**	**Forensic hospitals (%)**	**General psychiatric hospitals (%)**	**Forensic hospitals (%)**	**General psychiatric hospitals (%)**
2015	2.0	0.6	0.4	0.2	1.5	0.4
2016	2.8	0.6	1,8	0.4	1.2	0.2
2017	1.9	0.7	0.9	0.4	1.1	0.3

In the 32 general psychiatric hospitals, 115,011 patients were treated in 2017 (Table 1) with a total of 3,178,828 days of treatment. In general psychiatry, 3,281 cases (2.9%) were subjected to 9,716 seclusion episodes and 5,421 cases (4.7%) were subjected to 17,131 restraint episodes (Table 3). The mean cumulated duration of seclusion episodes per affected case was 32.2 h (Table 4) and the mean cumulated duration of restraint episodes was 37.6 h (Table 5). The mean total duration of the respective coercive intervention in relation to the total duration of hospital stay was 0.2% for seclusion episodes and 0.3% for restraint episodes. Involuntary medication was applied in 734 cases (0.7%, Table 6). The mean number of all coercive measures per bed per year was 3.1, 1.1 for seclusion and 2.0 for restraint. The mean number of involuntary medications was 0.07 for emergency medication and 0.06 for medication according to a court order.

## Discussion

To our knowledge, this is the first publication reporting the use of coercive interventions in forensic psychiatric services for a politically defined catchment area over several years. Moreover, the case registry offers the possibility to compare the obtained results with data on all general psychiatric hospitals in the same Federal State of 11 million inhabitants, recorded by the same methods, and under identical definitions of coercive interventions. Due to strict legal requirements, repeated conferences on data quality among all hospitals run by the Ministry of Social Welfare and Integration, and the necessity to submit raw data collected in electronic charts to the registry, the validity of the data is probably good to very good (5). The results indicate that nearly one out of four (22.6%) of the treated cases in forensic psychiatric facilities were subjected to seclusion during a year of detention, while physical restraint concerned less than one out of 25 cases (3.6–3.8%). The proportion of patients subjected to seclusion was about 8-fold higher than among patients in general psychiatric hospitals, while the proportion of patients subjected to mechanical restraint was slightly lower. Also the cumulative duration of seclusion episodes was 6- to 9-fold higher in the reported years, comparing the median values which can be considered as relatively robust against outliers of extreme cases. The use of involuntary medication was about 3-fold higher in forensic psychiatric hospitals, but still on a low level, never exceeding 3% of treated cases. From the hospitals' point of view, however, forensic institutions have fewer coercive medications per bed per year than general psychiatric hospitals. Variations between the years were generally small, and considerably smaller in general psychiatric hospitals, probably due to the higher number of cases. Room confinement was only used in forensic psychiatric facilities and concerned about one in eight cases.

The result of the higher use of coercive interventions in forensic psychiatric facilities is not unexpected and indicates a higher degree of risk of violence toward others as all patients in forensic hospitals have been detained by criminal courts following significant offenses. Yet our data do not allow to draw conclusions on whether these differences can be wholly explained by the different patient characteristics or whether different institutional practices and cultures also play a role.

An interesting topic of discussion between forensic psychiatrists and general psychiatrists is the different use of seclusion and mechanical restraint. In forensic psychiatry, mechanical restraint is used very rarely, which is not the case in general psychiatry. The reason, according to discussions among clinicians, is that the duration of these interventions is time limited in most cases in general psychiatry, with a mean duration of a single measure of about 8 h (6), indicated by a cumulative duration per case of about 40 h (Table 5). During mechanical restraint with 1:1 supervision, this period is used for building a relationship with the patient and trying to make agreements related to medication and non-violent behavior. Moreover, some of the patients are intoxicated at the beginning of mechanical restraint, and medical controls such as blood pressure controls are considered necessary. In contrast, many patients in forensic psychiatry are agitated and dangerous for considerably longer periods of time, but usually they are not intoxicated and not in a critical medical condition. Using mechanical restraint instead of seclusion for periods of not hours but days or even weeks (as indicated by the data presented in Table 5) would be considered as inappropriate restriction and the use of mechanical restraint is restricted to dangerous, self-injurious behavior.

In comparison with general psychiatry, it is noticeable that relatively few cases are treated in forensic psychiatry (1.2% of the general psychiatric case numbers). However, these cases account to 11.5% of the number of treatment days in general psychiatry, reflecting the selection of clinically severe cases in forensic psychiatry.

A comparison of the eight different forensic psychiatric hospitals, as we did for the general psychiatric hospitals (7), makes little sense, since the mandate by law and thus the diagnoses differ between the hospitals and some of the most dangerous patients are transferred from the other hospitals to one specialized facility. From an epidemiological perspective, the data are only conclusive if analyzed on an entire Federal State-level as we did here.

Our study has some limitations. First, there exists no external validation in the strict sense.

So even if very unlikely, given the background of legal obligations, underreporting cannot completely be excluded. This refers particularly to the approval of involuntary medication by a judge, which could be omitted from the recording process on the form sheet for coercive measures after the long run of legal procedures. But there is a degree of control, after all, as in the case of eye-catching values, inquiries are made by the commissioned office and the validity of the data is thoroughly checked by the respective hospitals.

Second, there is no data available on the reasons why coercive measures were carried out.

As data privacy has a high priority in Germany, only limited data may be gathered on the central register. Yet the hospitals themselves gather vast amounts of information on the reasons and consequences of coercive interventions. The data from the central register is discussed annually among representatives of the hospitals at a specialist conference which frequently results in close examinations of attendant circumstances of coercive interventions.

Third, is the decision to refer an individual to forensic psychiatry and to discharge someone later on, depends on local courts' decisions and not on clinical judgment. Therefore, forensic psychiatric populations in other Federal states of Germany might be somewhat different.

Fourth, our study is based on retrospectively collected routine data and uses only aggregated data for secondary analyses. However, retrospective data collection is used in all studies on coercive measures where a researcher is not present fulltime to observe what happens. Real prospective studies in a strict sense are extremely rare in the field.

Due to the wide lack of comparable data from other countries, it is difficult to interpret the results in terms of appropriateness or quality. Some comparable material can be found in two publications from one of the two forensic psychiatric hospitals in Finland. Putkonen et al. (8) evaluated an intervention in a hospital with 13 wards and reported an amount of about 100 h in seclusion per 100 patient days over the wards and different periods. From this result, it can be calculated that on average patients spent about 4.2% of their stay in seclusion. In another study from the same forensic hospital in Finland (9), the incidence of seclusion was indicated as 47.7–58.4 days per 1,000 patient days, which would mean that patients spent about 5% of their stay in seclusion. In comparison, patients in our facilities seem to have spent less time of their stay in freedom-restrictive measures, which amounts to 2.7% if seclusion, restraint, and room confinement are added together.

However, the low prevalence of involuntary medication, compared to the high prevalence of freedom-restrictive measures needs in-depth discussion. Involuntary medication, if applied as a rapid tranquilizing injection, may often be connected with feelings of shame and humiliation (10), all the more so if basic rules of sensitive handling are not adhered to as much as would be desirable.

Nevertheless, in our opinion, adequate medication treatment of acute crises could reduce restraint and seclusion and the total amount of coercion. The major reason for the low prevalence of involuntary medication is the high threshold of legal requirements for treatment against a patient's will to be approved by a judge. Though over 40% of the cases had a diagnosis of a psychotic disorder (schizophrenia or mania), only in about 2% of cases involuntary medication was approved. It is improbable and in contrast to findings from patient interviews (11) that all the other patients in forensic psychiatry take prescribed medication on a voluntary basis. Rather, a certain proportion remains untreated and is subjected to freedom-restrictive measures which may be necessary to cope with psychosis-driven dangerous behavior. However, some of the patients are therapy-resistant under all treatment regimes of antipsychotics and not all episodes of seclusion could be prevented or shortened by the use of medication. Seclusion and restraint, in contrast to medication, do not constitute treatment and do not improve underlying psychotic states, which is reflected in “outliers” with overly long duration of seclusion. Findings from a randomized controlled study (12) suggest that the risk of being secluded can be roughly halved if medication is used preferably and observation studies suggest that the subjective burden of distress from involuntary medication is less than the distress from seclusion for most patients (13, 14).

In the Netherlands, formerly very high rates of seclusion dropped considerably after increased use of medication (15, 16). Regarding the treatment of people with schizophrenic and manic disorders, such as in-patients in German general psychiatry, we found that seclusion, restraint, and violent incidents increased considerably in a period when involuntary medication was not admissible due to a legal gap and then, following revised legislation, decreased to the previous level (17). Therefore, we have serious concerns about whether the high legal threshold to obtain permission for the use of medication against a patient's expressed will, also among those without capacity to consent, really represents an empowerment of patients' human rights. It seems to be compensated by a significant loss of freedom in terms of prolonged seclusion for many patients. There are therefore grounds to critically reflect on the current legal situation.

## Data Availability Statement

The datasets generated for this study will not be made publicly available. The data stored in the register is classified as confidential by the data protection officer of Baden-Wuerttemberg and is not publicly available due to data privacy.

## Ethics Statement

The Ethics Committee of Ulm University waived the requirement for ethics approval as approval is not required for studies analyzing anonymized data, in accordance with national legislation and institutional requirements.

## Author Contributions

EF made the evaluations and calculations and contributed to introduction, methods, and discussion. TS and UF contributed substantially to introduction and discussion.

### Conflict of Interest

TS and EF are commissioned with the operation of the central register for coercive measures. The remaining author declares that the research was conducted in the absence of any commercial or financial relationships that could be construed as a potential conflict of interest.
